# Optical imaging reveals chemotherapy-induced metabolic reprogramming of residual disease and recurrence

**DOI:** 10.1126/sciadv.adj7540

**Published:** 2024-04-05

**Authors:** Enakshi D. Sunassee, Riley J. Deutsch, Victoria W. D’Agostino, Pol Castellano-Escuder, Elizabeth A. Siebeneck, Olga Ilkayeva, Brian T. Crouch, Megan C. Madonna, Jeffrey Everitt, James V. Alvarez, Gregory M. Palmer, Matthew D. Hirschey, Nirmala Ramanujam

**Affiliations:** ^1^ Department of Biomedical Engineering, Duke University, Durham, NC, USA.; ^2^ Duke Molecular Physiology Institute and Sarah W. Stedman Nutrition and Metabolism Center, Durham, NC, USA.; ^3^ Department of Pharmacology and Cancer Biology, School of Medicine, Duke University, Durham, NC, USA.; ^4^ Department of Medicine, Division of Endocrinology, Metabolism, and Nutrition, Duke University Medical Center, Durham, NC, USA.; ^5^ Duke University Trinity College of Arts and Sciences, Durham, NC, USA.; ^6^ Department of Pathology, School of Medicine, Duke University, Durham, NC, USA.; ^7^ Public Health Sciences Division, Fred Hutchinson Cancer Center, Seattle, WA, USA.; ^8^ Department of Radiation Oncology, Duke University, Durham, NC, USA.

## Abstract

Fewer than 20% of triple-negative breast cancer patients experience long-term responses to mainstay chemotherapy. Resistant tumor subpopulations use alternative metabolic pathways to escape therapy, survive, and eventually recur. Here, we show in vivo, longitudinal metabolic reprogramming in residual disease and recurrence of triple-negative breast cancer xenografts with varying sensitivities to the chemotherapeutic drug paclitaxel. Optical imaging coupled with metabolomics reported an increase in non–glucose–driven mitochondrial metabolism and an increase in intratumoral metabolic heterogeneity during regression and residual disease in resistant MDA-MB-231 tumors. Conversely, sensitive HCC-1806 tumors were primarily reliant on glucose uptake and minimal changes in metabolism or heterogeneity were observed over the tumors’ therapeutic life cycles. Further, day-matched resistant HCC-1806 tumors revealed a higher reliance on mitochondrial metabolism and elevated metabolic heterogeneity compared to sensitive HCC-1806 tumors. Together, metabolic flexibility, increased reliance on mitochondrial metabolism, and increased metabolic heterogeneity are defining characteristics of persistent residual disease, features that will inform the appropriate type and timing of therapies.

## Teaser

Optical imaging reveals differences in metabolic heterogeneity over the life cycle of paclitaxel-resistant and -sensitive tumors.

## INTRODUCTION

Triple-negative breast cancer (TNBC) tumors lack detectable estrogen receptor (ER), progesterone receptor (PR), and human epidermal growth factor receptor 2 (HER2) gene amplification (*1*). Therefore, widely used targeted therapies are ineffective for TNBC patients, rendering chemotherapy the main therapeutic option (*2*, *3*). TNBC patients treated with cytotoxic therapies such as paclitaxel or gemcitabine record a response rate of less than 20% (*2*, *4*, *5*); therefore, recurrence rates of these tumors remain high (*3*, *6*). Residual cells that escape therapy represent a reservoir of cancer cells that can lead to recurrence. These cancer cells survive extreme conditions such as therapy-induced stress, persist undetected and asymptomatic for years in a quiescent state, and may recur when conditions are favorable (*7*). Therefore, studying residual disease will inform therapeutic strategies aimed at reducing or delaying tumor recurrences.

Residual cell survival and re-activation are selective processes that are directly affected by the tumor microenvironment (*7*). Therefore, metabolic characteristics of residual disease may not be a simple shift from one phenotype to another; rather, distinct subpopulations could give rise to different metabolic phenotypes consisting of either aerobic or anaerobic respiration (*8*, *9*). Recent studies have shown that therapy-resistant TNBC tumors rely on mitochondrial metabolism and additional energetic sources (*10*) including amino acids, such as glutamine, and lipids, such as palmitate, during metabolic stress (*11*, *12*). However, conclusions drawn from studies investigating tumor metabolism depend on the tumor model used, the time points at which they are studied, and the metabolic features that are characterized. Additionally, many detailed metabolic studies on TNBC chemoresistance are limited to in vitro or excised samples (*13*–*16*) and therefore are unable to report on long-term persistence of residual disease subpopulations and their propensity for recurrence.

We have developed an optical microscopy platform for the spatiotemporal imaging of major metabolic pathways in vivo (*17*). Specifically, we have extensively validated the use of fluorescent reporters 2-[*N*-(7-nitrobenz-2-oxa-1,3-diazol-4-yl) amino]-2-deoxy-d-glucose (2-NBDG), tetramethylrhodamine ethyl ester (TMRE), and Bodipy FL C16 to report on glucose uptake, mitochondrial membrane potential, and fatty acid uptake, respectively (*18*, *19*). We have validated TMRE measurements to provide inference of mitochondrial metabolism in situ through studies investigating the effect of mitochondrial decouplers and oxygen depletion on TMRE uptake and quantifying metabolites in the citric acid cycle (*18*, *20*). Here, we report on metabolic reprogramming of clinically relevant models of treatment-resistant and treatment-sensitive TNBC tumors over their entire therapeutic life cycle (*21*, *22*). Treatment-resistant MDA-MB-231 tumors show a strong shift from predominantly glucose metabolism to mitochondrial metabolism following paclitaxel therapy, with the presence of intratumoral residual subpopulations that rely on glucose uptake, mitochondrial metabolism, or both. In stark contrast, treatment-sensitive HCC-1806 tumors show minimal metabolic plasticity. Treatment-resistant HCC-1806 tumors show elevated mitochondrial metabolism and intratumoral heterogeneity compared to sensitive HCC-1806 tumors. Results describing changes in metabolic heterogeneity throughout this article refer to differences in the metabolic preferences of tumor subpopulations on mitochondrial metabolism and glucose uptake either within (intra-) or between (inter-) tumors. Together, these results show that increased plasticity and intratumoral heterogeneity are defining features of metabolic reprogramming in residual disease and recurrence.

## RESULTS

### Metabolic plasticity of resistant MDA-MB-231 tumors

**Fig. 1.**
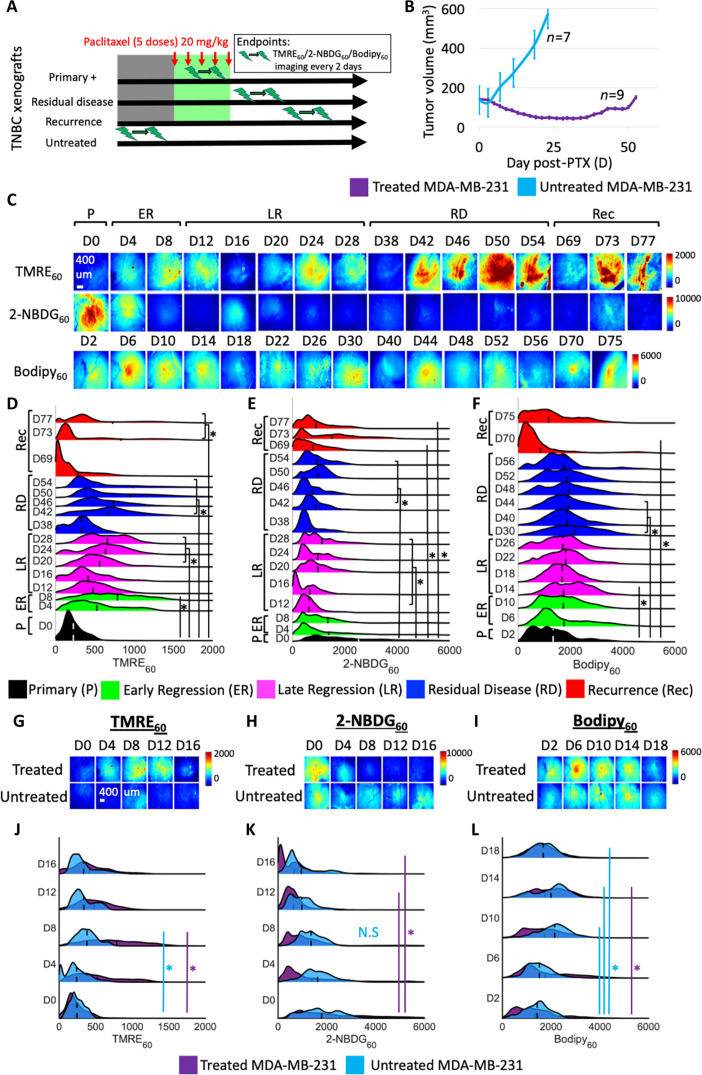
Resistant MDA-MB-231 tumors show metabolic plasticity over their therapeutic life cycle. (**A**) MDA-MB-231 xenografts were treated with paclitaxel under a maximum dose density regimen. Mitochondrial metabolism (TMRE), glucose uptake (2-NBDG), and fatty acid uptake (Bodipy FL C16) were imaged in vivo. Xenografts showed an initial response to paclitaxel, a period of residual disease, and a resurgence in tumor volume at ~60 days after drug withdrawal. (**B**) Mean tumor volumes (mm^3^) where D0 indicates the start of treatment. Error bar, SE (*n* = 9). (**C**) Representative images of primary (P) (*n* = 10), early regressing (ER) (*n* = 10), late regressing (LR) (*n* = 10), residual disease (RD) (*n* = 10), and recurring (Rec) (*n* = 13) MDA-MB-231 tumors. Ridge plots across all pixels and all treated mice show (**D**) an increase in mitochondrial metabolism, (**E**) a decrease in glucose uptake, and (**F**) a slight increase in fatty acid uptake at key time points during RD compared to the primary tumor (*P* < 0.05). Representative images of (**G**) mitochondrial metabolism, (**H**) glucose uptake, and (**I**) fatty acid uptake of untreated (*n* = 5) and treated (*n* = 10) tumors every 2 days up to the tumor burden limit. Ridge plots of probe uptake across all pixels of all untreated mice at each time point show (**J**) an increase in mitochondrial metabolism at one time point (*P* < 0.05: D8 versus D0), (**K**) no significant temporal changes in glucose uptake (*P* > 0.05), and (**L**) an increase in fatty acid uptake at three time points (*P* < 0.05: D0 versus D10, D14, and D18). Statistical differences in probe uptake were determined using a KS test. Vertical dashed lines superimposed on each curve correspond to the average fluorescence. For bracketed time points, each individual time point is significantly different from the primary tumor (D0). The representative image is consistent with trends seen in the average fluorescence across all mice at each time point.

We sought to quantify in vivo longitudinal metabolic reprogramming during regression, residual disease, and recurrence in MDA-MB-231 xenografts treated with the chemotherapeutic drug paclitaxel. Following orthotopic injection with MDA-MB-231 human TNBC cells in female athymic nudes, tumors were allowed to reach a dimension of ~150 mm^3^ and are referred to as “primary tumors.” Once tumors reached this volume, mice were administered paclitaxel under a conventional maximal dose density regimen [five intraperitoneal doses of 20 mg/kg each, on day 0 (D0), D2, D4, D6, and D8]. Metabolic imaging was performed every 2 days to capture subtle changes following paclitaxel therapy. Each TMRE, 2-NBDG, or Bodipy FL C16 endpoint was captured 60 min after injection of the respective probe based on our previously validated probe uptake kinetics and is indicated by the summary parameters 2-NBDG_60_, TMRE_60_, and Bodipy_60_ (*17*, *19*). Imaging was performed until the end of the viability of the window chambers (~14 days) or until the tumor burden limit was reached (Fig. 1A).

MDA-MB-231 tumors (*n* = 9) showed a ~30% volume reduction 4 days after initiation of paclitaxel (“early regression”) after two doses of paclitaxel, a rate that is consistent with that shown in the literature and comparable to clinical timelines (Fig. 1B) (*21*). Tumor volume continued to shrink after treatment with a ~60% reduction 18 days after initiation of paclitaxel (“late regression”), and no active growth was seen throughout the “residual disease” stage (*23*), which occurred ~40 days after the initiation of treatment. Spontaneous regrowth (“recurrence”) occurred at ~60 days, although this timeline varied by a few days across individual tumors. Conversely, untreated tumors displayed an exponential growth until the tumor burden limit (*n* = 7).

MDA-MB-231 tumors showed a strong metabolic shift during their therapeutic life cycle (Fig. 1C). Glucose uptake (2-NBDG_60_) decreased during disease regression, residual disease, and recurrence compared to untreated/primary tumors, whereas mitochondrial metabolism (TMRE_60_) was elevated in post-treatment tumors. Fatty acid uptake (Bodipy_60_) showed a slight increase during the tumor regression and residual disease stages, and a decrease during the tumor recurrence stage compared to untreated/primary tumors.

The probability density functions (PDFs) of each metabolic probe (across all mice at each time point) are shown as ridgeline plots (Fig. 1, D to F). Scatterplots show changes in average probe uptake for each individual MDA-MB-231 tumor over time (fig. S1, A to C). There was a significant increase in mitochondrial metabolism as early as 2 days after the third paclitaxel dose [*P* < 0.05, Kolmogorov-Smirnov (KS) test] in the early regression stage (*n* = 10), which was sustained during late regression (*n* = 10) and residual disease (*n* = 10) (*P* < 0.05, KS test) (Fig. 1D). There was a significant decrease in glucose uptake as early as 2 days after the fifth paclitaxel dose in the early regression stage, which was maintained during the late regression, residual disease, and recurrence (*n* = 13) stages (*P* < 0.05, KS test) (Fig. 1E). Conversely, there was a slight increase in fatty acid uptake during early regression, late regression, and residual disease (*P* < 0.05 at four time points shown by an “*”; KS test) (Fig. 1F). Recurrent tumors had significantly decreased fatty acid uptake compared to primary tumors (*P* < 0.05, KS test). Only two Bodipy_60_ measurements were captured at the recurrence time point compared to three TMRE_60_ and 2-NBDG_60_ measurements, respectively, since the recurrent tumors reached the tumor burden limit before the third Bodipy FL C16 imaging session. Imaging experiments at the primary, regression, and residual disease time points were carried out in two independent trials of *n* = 5 mice for a total *n* = 10 per imaging time point. Imaging experiments at the recurrence time point were carried out in two independent trials of *n* = 6 and *n* = 7 mice for a total *n* = 13. Additional mice were ordered in case of unexpected deaths during the study. If there were no unexpected deaths, these mice were imaged at the recurrence time point, accounting for a slightly larger sample size.

To confirm that metabolic changes were a result of paclitaxel treatment, we sought to capture longitudinal metabolic changes in untreated MDA-MB-231 tumors up to the tumor burden limit (*n* = 5 per imaging probe). Metabolic changes over time were compared to that of treated mice. It should be noted that the D0 time point for both the treated and untreated groups corresponds to untreated tumors. Representative images show that mitochondrial metabolism increased (Fig. 1G), while glucose uptake decreased over time in treated compared to untreated tumors (Fig. 1H). Fatty acid uptake was comparable between the two groups (Fig. 1I).

Analysis of ridgeline plots confirms the qualitative differences in 2-NBDG_60_ (Fig. 1H) observed in untreated tumors (D0 to D16) (*P* > 0.05, KS test) (Fig. 1, J and K) throughout the imaging time course. An increase in mitochondrial metabolism was observed in untreated tumors on D8 relative to D0 (*P* < 0.05, KS test); however, this was significantly less pronounced than in treated mice at the same time point (*P* < 0.05, KS test) (Fig. 1, J and K), supporting the qualitative observations (Fig. 1G). Significant increases in fatty acid uptake were observed on D14 in treated and on D10, D14, and D18 in untreated mice relative to that of the primary tumor at D0 (*P* < 0.05, KS test) (Fig. 1L). Individual statistical comparisons for all ridgeline plots are reported in Fig. 1.

To further confirm that metabolic changes were a result of paclitaxel treatment, normal female athymic nudes (no tumor) were treated with paclitaxel under a similar maximum dose density regimen. Mice were imaged at time points corresponding to the primary (D0), early regression (D4), late regression (D24), and residual disease (D42) stages in treated MDA-MB-231 mice. Although increases in glucose uptake were seen over time in paclitaxel-treated non–tumor-bearing mice, corresponding to typical mammary gland development, metabolic changes observed in treated MDA-MB-231 tumors were not observed (*24*–*27*) (fig. S2). Together, MDA-MB-231 tumors treated with paclitaxel show an increase in mitochondrial metabolism, a decrease in glucose uptake, and a slight increase in fatty acid uptake during disease regression and residual disease.

### Metabolic inflexibility of sensitive HCC-1806 tumors

**Fig. 2.**
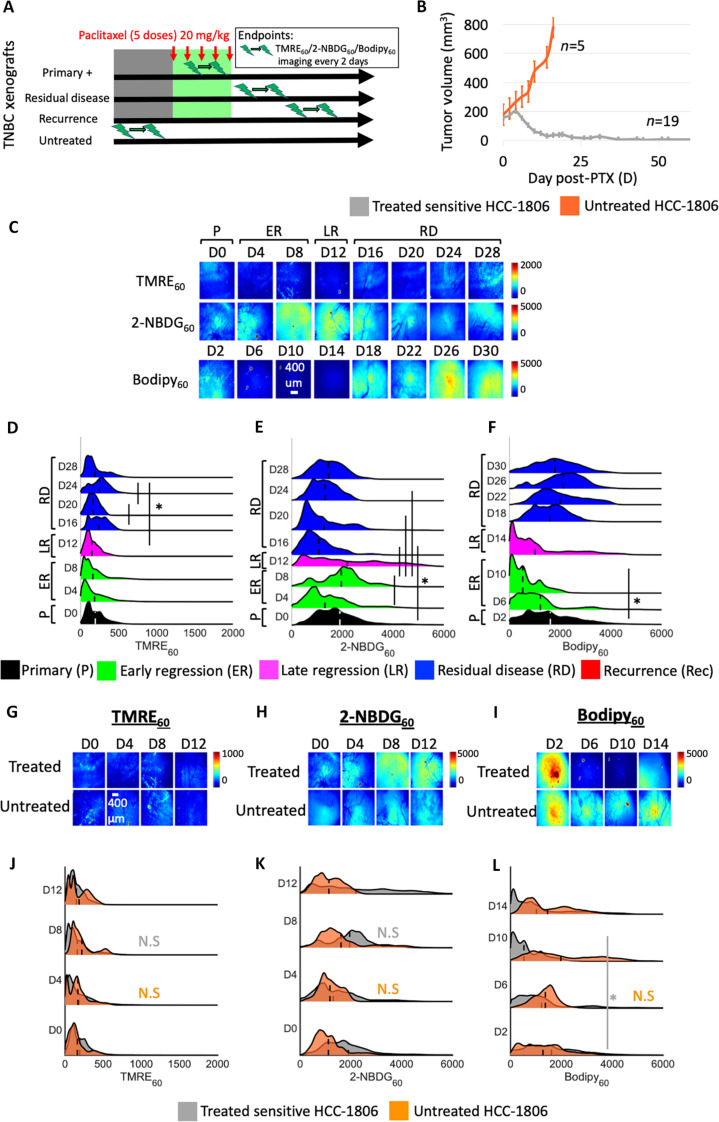
Sensitive HCC-1806 tumors are metabolically inflexible over their therapeutic life cycle. (**A**) HCC-1806 xenografts were treated with paclitaxel under a maximum dose density regimen when tumor sizes reached ~150 mm^3^. Mitochondrial metabolism (TMRE), glucose uptake (2-NBDG), and fatty acid uptake (Bodipy FL C16) were imaged in vivo. Sensitive xenografts showed no evidence of residual disease up to 120 days after treatment withdrawal. (**B**) Mean tumor volumes (mm^3^) where D0 indicates the start of paclitaxel treatment. Error bar, SE (*n* = 19). (**C**) Representative images of primary (P) (*n* = 10), early regressing (ER) (*n* = 10), late regressing (LR) (*n* = 10), and residual disease (RD) (*n* = 10) HCC-1806 tumors. Ridge plots of (**D**) mitochondrial metabolism, (**E**) glucose uptake, and (**F**) fatty acid uptake across all pixels and all treated mice at each time point. Representative images of (**G**) mitochondrial metabolism, (**H**) glucose uptake, and (**I**) fatty acid uptake of untreated (*n* = 5) and treated (*n* = 10) tumors every 2 days up to the tumor burden limit. Ridge plots of probe uptake across all pixels and all mice at each time point show no significant changes in (**J**) mitochondrial metabolism, (**K**) glucose uptake, or (**L**) fatty acid uptake (*P* > 0.05) in untreated mice. Statistical differences in probe uptake were determined using a KS test. Vertical dashed lines superimposed on each curve correspond to the average fluorescence. An asterisk, “*,” signifies statistical significance between two time points joined by the corresponding line. The representative image is consistent with trends seen in the average fluorescence across all mice at each time point.

We next sought to interrogate metabolic changes in a second TNBC xenograft model where heterogeneous responses to paclitaxel treatment were seen within the same tumor line, i.e., a case where some mice recurred (resistant) and some mice did not (sensitive), despite being treated at the same initial tumor volume. HCC-1806 xenografts (*n* = 19) were treated with paclitaxel using a similar approach to MDA-MB-231 mice (Fig. 2A). Untreated tumors displayed an exponential growth until the tumor burden limit was reached (Fig. 2B). Consistent with the literature, 60% of the mice in the treated group showed long-term sensitivity to paclitaxel, with no palpable tumor at 120 days after paclitaxel withdrawal (termed sensitive HCC-1806) (*n* = 19) (Fig. 2B) (*22*). This section will focus on the metabolic response of sensitive HCC-1806 tumors. A comparison of the metabolism between day-matched resistant and sensitive tumors will be discussed later in the article.

Representative metabolic images of sensitive HCC-1806 tumors showed an increase in glucose uptake (2-NBDG_60_) during and immediately after paclitaxel treatment (Fig. 2C), but glucose uptake returned to baseline levels during late tumor regression and residual disease. Mitochondrial metabolism (TMRE_60_) was unchanged during disease regression and residual disease. Although fatty acid uptake (Bodipy_60_) decreased during tumor regression, this change was transient, resolving itself at the residual disease stage. The PDFs of each metabolic probe are shown as ridgeline plots (Fig. 2, D to F). Scatterplots show changes in average probe uptake for each individual HCC-1806 tumor over time (fig. S1, D to F). No significant changes in mitochondrial metabolism were observed throughout the course of paclitaxel treatment (*n* = 10) (*P* > 0.05) (Fig. 2D). A nonsignificant increase in glucose uptake was observed during early regression (*n* = 10) and late regression (*n* = 10) (*P* > 0.05) (Fig. 2E) followed by a significant decrease at the residual time points relative to the regression time points (*n* = 10) (*P* < 0.05, KS test). A transient decrease in fatty acid uptake was observed during early and late regression compared to untreated/primary HCC-1806 tumors, which was resolved during the residual disease stage (*P* < 0.05, KS test) (Fig. 2F).

To confirm that metabolic changes were a result of paclitaxel treatment, we sought to capture longitudinal metabolic changes in untreated mice beyond the primary time point only. Untreated HCC-1806 tumors were imaged longitudinally up to the tumor burden limit (*n* = 5 per imaging probe). Metabolic changes over time were compared to that of treated mice. It should be noted that the D0 time point for both the treated and untreated groups corresponds to untreated tumors. Representative images show comparable metabolic profiles in untreated mice over the course of imaging (D0 to D12) (Fig. 2, G to I). Analysis of ridgeline plots confirms no significant changes in any of these metabolic indicators over time in untreated tumors (D0 to D12) (*P* > 0.05, KS test) (Fig. 2, J to L). Therefore, sensitive, treated HCC-1806 tumors display only transient changes in glucose and fatty acid uptake during and immediately following acute treatment.

### Increased mitochondrial metabolism during regression and residual disease in resistant MDA-MB-231 tumors

**Fig. 3.**
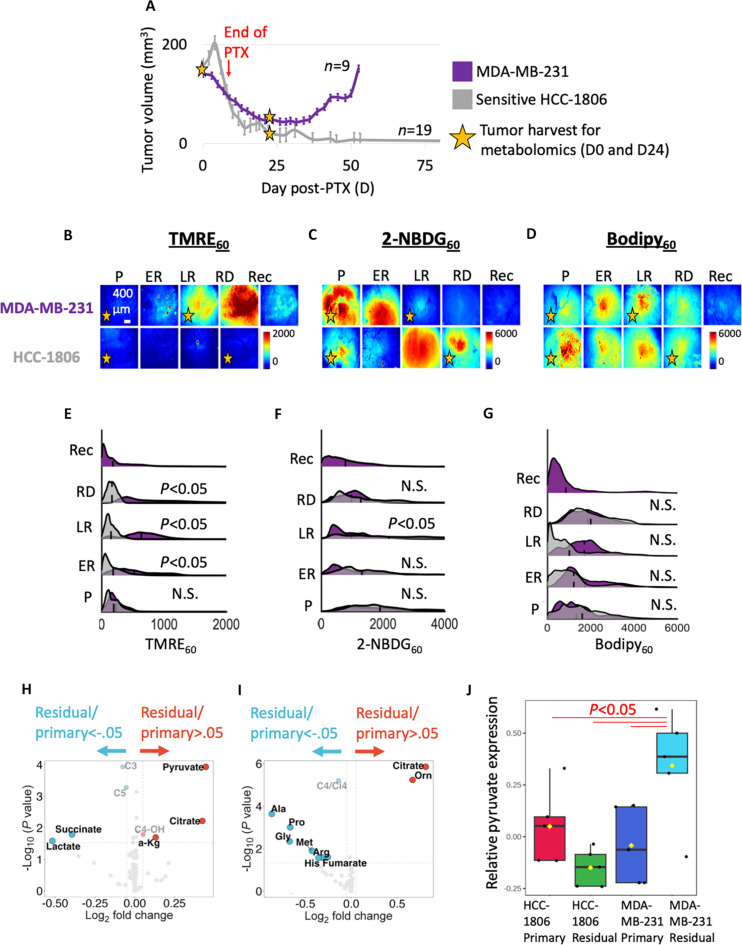
Imaging and metabolomics confirm increased mitochondrial metabolism during regression and residual disease in resistant MDA-MB-231 tumors. (**A**) Mean tumor volumes (mm^3^) where D0 indicates the start of paclitaxel treatment. MDA-MB-231 xenografts showed an initial response to paclitaxel, followed by residual disease, and tumor recurrence ~60 days after paclitaxel treatment, while sensitive HCC-1806 xenografts showed sustained responses to paclitaxel with no palpable tumor at 120 days. Error bar, SE (*n* = 9 MDA-MB-231; *n* = 19 HCC-1806). Representative images show (**B**) mitochondrial metabolism (TMRE_60_), (**C**) glucose uptake (2-NBDG_60_), and (**D**) fatty acid uptake (Bodipy_60_) of primary (P) (*n* = 10), early-regressing (ER) (*n* = 10), late-regressing (LR) (*n* = 10), residual disease (RD) (*n* = 10) HCC-1806 and MDA-MB-231 tumors, and recurring (Rec) MDA-MB-231 tumors (*n* = 13). Ridge plots show the probability density distribution of (**E**) mitochondrial metabolism, (**F**) glucose uptake, and (**G**) fatty acid uptake across all pixels and all mice at each time point. Statistical differences in probe uptakes were determined using a KS test. Vertical dashed lines superimposed on each curve correspond to the average fluorescence. (**H**) Volcano plot of fold change in metabolite levels between regressing and primary MDA-MB-231 tumors (*n* = 5 per group). (**I**) Volcano plot of fold change in metabolite levels between regressing and primary HCC-1806 tumors (*n* = 5 per group). Dashed lines represent *P* < 0.05 via a two-sided *t* test and | Log_2_FoldChange | > 0.05. Significantly different metabolites are labeled within the graph. (**J**) Bar plot of change in pyruvate levels between regressing and primary time points across MDA-MB-231 and HCC-1806 tumors (*n* = 5). Metabolite levels were normalized to account for batch effects across cell lines. Statistical differences in pyruvate levels were determined using a one-way ANOVA followed by Tukey’s post hoc test. The representative image is consistent with trends seen in the average fluorescence across all mice at each time point.

We compared the metabolic phenotypes of paclitaxel-resistant MDA-MB-231 and paclitaxel-sensitive HCC-1806 at the regression and residual disease time points (Fig. 3A). Representative images show that mitochondrial metabolism (TMRE_60_) was increased during regression and residual disease in MDA-MB-231 tumors, while minimal changes were observed over time in sensitive HCC-1806 tumors (Fig. 3B). Glucose uptake (2-NBDG_60_) decreased during regression, residual disease, and recurrence in MDA-MB-231 tumors, while a transient increase was observed in sensitive HCC-1806 tumors only at the regression time point (Fig. 3C). MDA-MB-231 tumors exhibited a slight increase in fatty acid uptake (Bodipy_60_) during the regression and residual disease time points, followed by a decrease at the recurrence time point. On the other hand, sensitive HCC-1806 tumors showed a decrease in fatty acid uptake during regression, which was resolved at the residual disease time point (Fig. 3D).

As seen in the ridge plots, no significant differences in mitochondrial metabolism (Fig. 3E), glucose uptake (Fig. 3F), or fatty acid uptake (Fig. 3G) were observed between the primary HCC-1806 and MDA-MB-231 tumors. However, mitochondrial metabolism was significantly higher, glucose uptake was significantly lower, and fatty acid uptake was comparable in MDA-MB-231 tumors compared to HCC-1806 tumors during regression and/or residual disease. Scatterplots show changes in average probe uptake for each individual MDA-MB-231 and HCC-1806 tumor at selected time points (fig. S1, G to I).

Next, we sought to interrogate metabolite distributions at key time points corresponding to the largest differences in metabolic changes from our imaging studies. Tumor harvest for metabolomics was day-matched across tumor lines to enable comparisons. Metabolomic analysis showed that tricarboxylic acid (TCA) cycle intermediates pyruvate, citrate, and α-ketoglutarate (α-KG) pools were significantly increased, while pools of the glycolytic intermediate lactate were significantly decreased in residual MDA-MB-231 tumors compared to primary tumors (*P* < 0.05 and |Log_2_FoldChange| > 0.05) (*n* = 5 tumors per time point) (Fig. 3H). Conversely, this trend in TCA cycle and glycolytic intermediates was not observed in residual HCC-1806 tumors compared to primary tumors, except for citrate (*n* = 5 tumors per time point) (Fig. 3I). Across cell lines, pyruvate was observed to be significantly higher in residual MDA-MB-231 tumors compared to all other groups (primary MDA-MB-231 tumors, primary and residual HCC-1806 tumors), pointing to oxidative phosphorylation (OXPHOS) as central to the mechanism underlying our observed up-regulation in mitochondrial metabolism (Fig. 3J). Our longitudinal imaging and metabolomic analyses demonstrate that resistant MDA-MB-231 tumors showed increased mitochondrial metabolism following treatment, while sensitive HCC-1806 tumors displayed minimal deviation in glycolytic and mitochondrial metabolism from their baseline phenotype following paclitaxel therapy.

### Increased intratumoral metabolic heterogeneity in resistant MDA-MB-231 tumors compared to sensitive HCC-1806 tumors

**Fig. 4.**
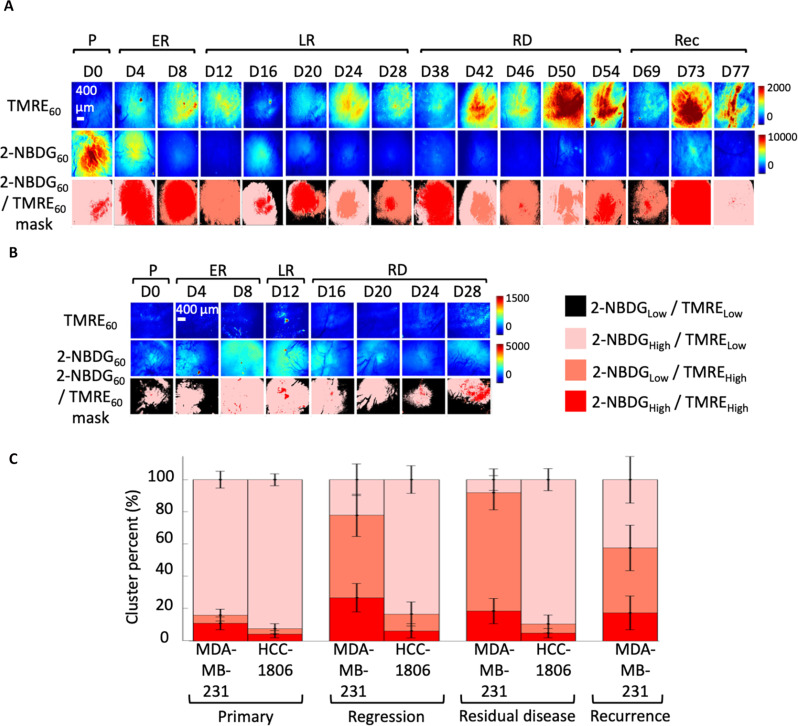
Resistant MDA-MB-231 tumors show increased intratumoral metabolic heterogeneity compared to sensitive HCC-1806 tumors. (**A**) Representative images of primary (P) (*n* = 10), early regressing (ER) (*n* = 10), late regressing (LR) (*n* = 10), residual disease (RD) (*n* = 10), and recurrence (Rec) (*n* = 13) for mitochondrial metabolism (TMRE_60_), glucose uptake (2-NBDG_60_), and the corresponding mitochondrial metabolism/glucose uptake (TMRE_60_/2-NBDG_60_) cluster mask in MDA-MB-231 tumors. (**B**) Representative images of primary (P) (*n* = 10), early regressing (ER) (*n* = 10), late regressing (LR) (*n* = 10), and residual disease (RD) (*n* = 10) for mitochondrial metabolism (TMRE_60_), glucose uptake (2-NBDG_60_), and the corresponding mitochondrial metabolism/glucose uptake (TMRE_60_/2-NBDG_60_) cluster mask in sensitive HCC-1806 tumors. (**C**) Bar graphs showing mean changes in area fraction [cluster percent (%)] of cluster distributions corresponding to [2-NBDG_High_/TMRE_Low_] clusters, [2-NBDG_High_/TMRE_High_] clusters, or [2-NBDG_Low_/TMRE_High_] clusters across primary, regression, residual disease, and recurrence (if applicable) for each tumor line. Statistical differences in cluster percent were determined using a one-way ANOVA followed by Tukey’s post hoc test.

We next investigated intratumoral metabolic heterogeneity. Given the ability to differentiate between paclitaxel-resistant and paclitaxel-sensitive models based on their mitochondrial metabolism (TMRE_60_) and glucose uptake (2-NBDG_60_) phenotypes, we sought to quantify spatial metabolic heterogeneity of these two metabolic endpoints using cluster analysis. First, we defined four metabolic clusters: low glucose uptake and low mitochondrial metabolism [2-NBDG_Low_/TMRE_Low_], high glucose uptake and high mitochondrial metabolism [2-NBDG_High_/TMRE_High_], low glucose uptake and high mitochondrial metabolism [2-NBDG_Low_/TMRE_High_], and high glucose uptake and low mitochondrial metabolism [2-NBDG_High_/TMRE_Low_]. Cluster assignment was performed on a pixel-by-pixel basis across all images corresponding to all mice at each key time point for both cell lines (all available pixels across all mice). The mean values of each metabolic indicator were used as cutoffs for segmentation into “high” or “low” clusters. The percentage of the cluster was the mean across all the imaged time points corresponding to a specific disease stage. For example, for disease regression in MDA-MB-231 mice, the mean was calculated for imaged time points over days D4 to D28. We also evaluated the median values of each probe as cutoffs. Irrespective of the cutoff methods used, the distribution of each spatial cluster at each time point across both cell lines was the same. The cluster distribution of MDA-MB-231 tumors varied across time points (Fig. 4A). The cluster distribution of HCC-1806 mice appeared comparable across time points (Fig. 4B). It should be noted that the 2-NBDG_60_/TMRE_60_ mask in Fig. 4 (A and B) accounts for probe uptake across both metabolic endpoints, and therefore, clusters reflect the relative uptake of both TMRE and 2-NBDG.

Next, we computed the area fraction of each spatial cluster within each image in each group. In resistant MDA-MB-231 tumors, the area fraction of [2-NBDG_High_/TMRE_Low_] clusters was significantly lower during the regression, residual disease, and recurrence time points relative to that of the primary tumor (22.1% during regression, 8.1% during residual disease, and 42.3% during recurrence compared to 84.0% during primary) [one-way analysis of variance (ANOVA) followed by Tukey’s post hoc test] (Fig. 4C). Conversely, the area fraction of [2-NBDG_Low_/TMRE_High_] clusters was significantly higher during the regression, residual disease, and recurrence time points of MDA-MB-231 tumors compared to that of the primary tumor (51.1% during regression, 73.4% during residual disease, and 40.2% during recurrence compared to 5.1% during primary) (one-way ANOVA followed by Tukey’s post hoc test) (Fig. 4C). The cluster distribution was not significantly different for any metabolic cluster in HCC-1806 tumors across primary, regressing, and residual disease time points (83.3% during regression and 89.5% during residual disease compared to 92.4% during primary for [2-NBDG_High_/TMRE_Low_] clusters) (Fig. 4C). Additionally, a significant difference in the area fraction of [2-NBDG_Low_/TMRE_High_] clusters was observed between the tumor lines at the regression and residual disease time points (51.1% in MDA-MB-231 compared to 10.4% in HCC-1806 during regression and 73.4% in MDA-MB-231 compared to 5.6% in HCC-1806 during residual disease) (one-way ANOVA followed by Tukey’s post hoc test). On the other hand, the area fraction of [2-NBDG_High_/TMRE_Low_] clusters was significantly lower in MDA-MB-231 compared to HCC-1806 tumors (22.1% for MDA-MB-231 compared to 83.3% for HCC-1806 during regression, and 8.1% for MDA-MB-231 compared to 89.5% in HCC-1806 during residual disease) (one-way ANOVA followed by Tukey’s post hoc test).

To demonstrate how intratumoral heterogeneity varies across cell lines and time points, we expanded our results shown in Fig. 4C to evaluate metabolic clusters on a per-mouse basis (fig. S3). We observed that metabolic heterogeneity between individual MDA-MB-231 mice was increased at the regression, residual, and recurrent disease stages compared to the primary stage. We performed histological analyses to confirm that metabolic heterogeneity was not due to variations in viability (figs. S4 and S5). MDA-MB-231 tumors were viable across all disease stages, with small foci of degeneration and necrosis in the late regression (*n* = 5) and recurrent stages (*n* = 5). HCC-1806 mice were also viable across all groups, although the histopathology of the masses was more variable as there was more necrosis in these tumors compared to MDA-MB-213 neoplasms. Viable tumor regions displayed staining in ATP5A1, a key subunit of the adenosine triphosphate (ATP) synthase complex, which is a surrogate marker for mitochondrial content. On the basis of a semiquantitative grading scale performed by our board-certified pathologist, the amount of necrosis between untreated and treated HCC-1806 tumors was significant (*n* = 4) (*P* < 0.05). The amount of necrosis between untreated and treated MDA-MB-231 tumors was not significant (*n* = 4) (*P* > 0.05). Our data suggest that minimal changes in necrosis were seen in MDA-MB-231 tumors despite the dynamic metabolic changes reported. Statistical significance in necrotic fraction was performed using a one-way ANOVA followed by Tukey’s post hoc test.

Since variability in 2-NBDG_60_ signal was seen in treated non–tumor-bearing mice over time (fig. S2), we sought to analyze more subtle differences between tumor and nontumor groups by quantifying intratumoral metabolic heterogeneity on a per-mouse basis (fig. S3). Treated MDA-MB-231 tumor subpopulations displayed an increased reliance on mitochondrial metabolism, while treated normal mice showed variable contributions of glucose uptake and mitochondrial metabolism over time within each mouse. The residual disease stage of MDA-MB-231 tumors was primarily reliant on mitochondrial uptake, whereas, in stark contrast, normal tissues and residual HCC-1806 tumors were characterized by high glucose uptake across all time points. We also performed a similar heterogeneity analysis for treated and untreated MDA-MB-231 and HCC-1806 tumors (fig. S6). We observed that oxidative clusters were significantly larger in area at D8, D12, and D16 compared to the D0 time point in treated MDA-MB-231 tumors (one-way ANOVA followed by Tukey’s post hoc test). Conversely, no significant changes over time were observed in any of the cluster combinations (highly oxidative, highly glycolytic, or both) in untreated MDA-MB-231 tumors or treated and untreated HCC-1806 tumors (one-way ANOVA followed by Tukey’s post hoc test).

Additionally, in a separate study in which TNBC xenografts were treated with paclitaxel, imaging cohorts with window chambers that were 2 weeks old and those that were newly implanted showed no statistical differences in metabolic endpoints at overlapping time points (fig. S7). Together, our analyses show that resistant MDA-MB-231 tumors display increased intratumoral metabolic heterogeneity compared to sensitive HCC-1806 tumors after treatment, with residual subpopulations showing reliance on mitochondrial metabolism, glucose uptake, or both.

### Elevated intratumoral metabolic heterogeneity in resistant HCC-1806 tumors compared to sensitive HCC-1806 tumors

**Fig. 5.**
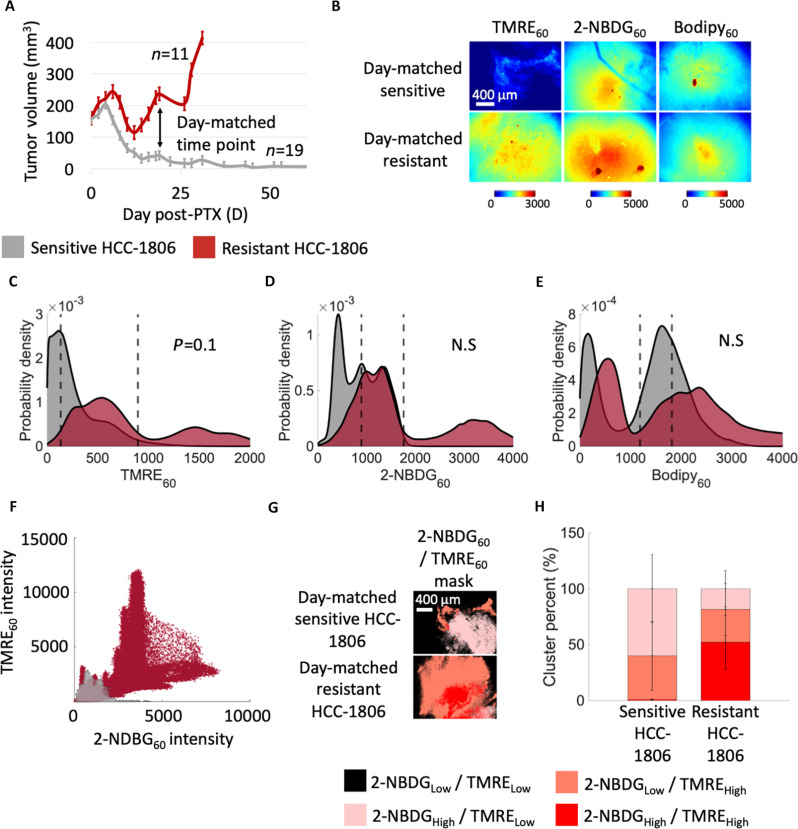
Resistant HCC-1806 tumors show elevated intratumoral metabolic heterogeneity compared to sensitive HCC-1806 tumors. HCC-1806 xenografts were treated with paclitaxel under a maximum dose density regimen when tumor sizes reached ~150 mm^3^. Xenografts showed a heterogeneous response to treatment, where some mice recurred and some did not. TMRE, 2-NBDG, and Bodipy FL C16 uptake were measured in vivo 60 min after injection in day-matched tumors that had high (recurred) or low (sensitive) tumor burdens. (**A**) Mean tumor volumes (mm^3^) where D0 indicates the start of paclitaxel treatment. Error bar, SE (sensitive HCC-1806 tumors, *n* = 19; resistant HCC-1806 tumors, *n* = 11). (**B**) Representative images of mitochondrial metabolism (TMRE_60_), glucose uptake (2-NBDG_60_), and fatty acid uptake (Bodipy_60_) for day-matched sensitive and resistant HCC-1806 tumors. Ridge plots show probability density distribution of (**C**) mitochondrial metabolism, (**D**) glucose uptake, and (**E**) fatty acid uptake across all pixels and all mice at each time point (*n* = 3, day-matched sensitive HCC-1806 tumors; *n* = 3, day-matched resistant HCC-1806 tumors). Vertical dashed lines superimposed on each curve correspond to the average fluorescence. (**F**) Scatterplots showing pixel-by-pixel distribution of glucose uptake versus mitochondrial metabolism (2-NBDG_60_ versus TMRE_60_ intensity) across sensitive HCC-1806 tumors and resistant HCC-1806 tumors. (**G**) Representative images of the representative TMRE_60_/2-NBDG_60_ cluster mask for day-matched sensitive and resistant HCC-1806 tumors. (**H**) Bar graphs show changes in area fraction [cluster percent (%)] across sensitive HCC-1806 tumors and resistant HCC-1806 tumors. Statistical differences in probe uptakes were determined using a KS test. Statistical differences in cluster percent were determined using a one-way ANOVA followed by Tukey’s post hoc test. The representative image is consistent with trends seen in the average fluorescence across all mice at each time point.

Next, we investigated differences in metabolism between HCC-1806 day-matched tumors that were sensitive and resistant to paclitaxel. Consistent with the literature, 60% of HCC-1806 mice showed long-term sensitivity to paclitaxel (*n* = 19), while 40% of the HCC-1806 mice were resistant to treatment (*n* = 11) (Fig. 5A), despite being treated at the same tumor volume (*22*). HCC-1806 mice that were resistant to treatment exhibited a significantly larger tumor burden than their sensitive counterparts during regression with no period of residual disease, enabling mice from these two groups to be distinguished as early as the “disease regression” phase based on their tumor volumes (~50% difference in tumor burdens between day-matched resistant and sensitive HCC-1806 tumors during early regression and ~78% difference in tumor burdens during late regression). Given that HCC-1806 tumors that underwent regression and residual disease had a different phenotype than those that recurred, we analyzed these two groups of mice separately and termed them sensitive HCC-1806 and resistant HCC-1806, respectively.

Day-matched tumors were imaged on D17 after initiation of paclitaxel, corresponding to ~78% difference in tumor burdens between resistant and sensitive HCC-1806 tumors. It should be noted that in this analysis, only day-matched HCC-1806 tumors were considered at D17 after initiation of paclitaxel, and therefore only represent a subset of the sensitive mice dataset used in the above analysis (Figs. 2 to 4). Nonetheless, trends in probe uptake and heterogeneity were consistent across both the original and the subset of sensitive HCC-1806 tumors.

Representative images show that fatty acid uptake (Bodipy_60_) and glucose uptake (2-NBDG_60_) were comparable in day-matched resistant and sensitive HCC-1806 tumors, suggesting comparable fatty acid and glucose uptake between both groups (Fig. 5B). Mitochondrial metabolism (TMRE_60_) was elevated in day-matched resistant HCC-1806 tumors compared to that of sensitive HCC-1806 tumors, suggesting an increase in oxidative metabolism (mean TMRE_60_ fluorescence = 136.9 ± 91.5 arbitrary unit (AU) in sensitive HCC-1806 mice and mean TMRE_60_ fluorescence = 896.7 ± 694.5 AU in resistant HCC-1806 mice).

Mitochondrial metabolism was nonsignificantly increased in day-matched resistant HCC-1806 tumors (*n* = 3) compared to sensitive HCC-1806 tumors (*n* = 3) (*P* = 0.1, KS test) (Fig. 5C). No significant changes in glucose uptake (*P* > 0.05, KS test) (Fig. 5D) or fatty acid uptake (*P* > 0.05, KS test) (Fig. 5E) were observed between the two groups. Additionally, resistant HCC-1806 tumors showed a wider distribution of both glucose uptake and mitochondrial metabolism (Fig. 5F), suggesting an increased propensity for tumors to be more metabolically active compared to their sensitive counterparts. A Shapiro-Wilk normality test of the primary HCC-1806 tumors confirmed a normal distribution, indicating that paclitaxel-resistant and paclitaxel-sensitive HCC-1806 tumors were indistinguishable at the primary time point based on their mitochondrial metabolism endpoints.

While the lack of significant differences in probe uptake may be due to the small sample size of each group (*n* = 3), we sought to further analyze metabolic heterogeneity, a critical feature associated with chemoresistance. We performed the same cluster assignment as previously described to identify regions of high glucose uptake, high mitochondrial metabolism, or both. The largest cluster in day-matched sensitive HCC-1806 mice corresponded to the [2-NBDG_High_/TMRE_Low_] cluster (Fig. 5G), suggesting an increased reliance on glycolysis relative to mitochondrial metabolism. Resistant HCC-1806 mice consisted of a higher proportion of [2-NBDG_High_/TMRE_High_] metabolic clusters compared to [2-NBDG_High_/TMRE_Low_] clusters (Fig. 5G), suggesting a reliance on both glycolysis and mitochondrial metabolism.

Next, we computed the area fraction of each spatial cluster within each image in each group. The resistant HCC-1806 mice reported an increase in the area fraction of [2-NBDG_High_/TMRE_High_] clusters compared to that of day-matched sensitive HCC-1806 mice (52.1% in day-matched resistant HCC-1806 mice and 0.67% in day-matched sensitive HCC-1806 mice) (one-way ANOVA followed by Tukey’s post hoc test) (Fig. 5H). Conversely, the resistant HCC-1806 mice reported a nonsignificant decrease in the area fraction of [2-NBDG_High_/TMRE_Low_] clusters compared to that of day-matched sensitive HCC-1806 mice (18.5% in day-matched resistant HCC-1806 mice and 60.1% in day-matched sensitive HCC-1806 mice) (one-way ANOVA followed by Tukey’s post hoc test) (Fig. 5H). Our comparisons show that within the heterogeneous HCC-1806 tumor line, tumors that recur display elevated mitochondrial metabolism and heterogeneity compared to tumors sensitive to paclitaxel.

## DISCUSSION

This study captured heterogeneous paclitaxel-induced changes along three metabolic axes (glucose uptake, mitochondrial membrane potential, and fatty acid uptake) following treatment in paclitaxel-resistant and paclitaxel-sensitive tumors (*17*). Additionally, longitudinal imaging pinpointed the timing of metabolic shifts associated with disease stage (regression, residual disease, and recurrence) and guided metabolomic analyses. Tumor landscape imaging captured metabolic heterogeneity—defined as an aggregate measure of the differences in the metabolic preferences of tumor subpopulations—associated with treatment resistance.

Resistant MDA-MB-231 tumors displayed a switch from glycolysis to mitochondrial metabolism following treatment, which was persistent throughout regression and residual disease. Intratumoral heterogeneity was characterized by regions of high glucose uptake, mitochondrial metabolism, or both, suggesting the potential of enhancing clinical outcomes by inhibiting metabolic signatures of residual disease. Further, our heterogeneity analyses (Figs. 4 and 5) show an increase in the area fraction of heterogeneous regions in MDA-MB-231 tumors and resistant HCC-1806 tumors compared to treatment-sensitive HCC-1806 tumors.

Evidence from the literature suggest that the presence of metabolically distinct tumor subpopulations ([2-NBDG_Low_/TMRE_High_] clusters in this study) could be driving recurrences. MYC- and MCL1-overexpressing TNBC stem cells showed increased mitochondrial metabolism, which further fueled stemness enrichment and induced paclitaxel resistance. Neoadjuvant chemotherapy-resistant TNBC tumors showed enrichment in stemness and amplification of an OXPHOS-dependent gene set (*28*). Further, enhanced fatty acid oxidation associated with stemness was seen in paclitaxel-resistant TNBC cells and fatty acid oxidation inhibition resulted in increased chemosensitivity. This mechanism suggests that recurrent TNBC tumors could have had an OXPHOS-dependent subpopulation of residual cells that drove recurrence (*29*). While we are unable to point to the exact mechanism of resistance without barcode-mediated clonal tracking, our data suggest that resistance mechanisms are multifaceted and are associated to the heterogeneous areas present in the tumor as supported by the TNBC literature (*4*).

We have previously shown that fatty acid oxidation inhibitor, etomoxir, pre-emptively blocked metabolic dependencies of residual tumors and prolonged tumor dormancy in a HER-2–driven genetically engineered mouse model of dormancy (*20*). Using residual TNBC patient-derived xenograft (PDX) tumor models, others have demonstrated that in vivo treatment with mitochondrial electron transport chain complex I inhibitor, IACS-10759, proved effective in improving long-term survival by targeting the chemotherapy-induced OXPHOS vulnerability (*13*). Future studies are warranted to investigate the chemical or genetic adaptive inhibition of mitochondrial metabolism during early regression following chemotherapy in clinically relevant TNBC PDX and patient-derived organoid (PDO) models exhibiting similar dependencies. This could inform on biomarkers of metabolic phenotypes and heterogeneity that can be used to guide future therapeutic decisions.

The metabolic switch observed in treated MDA-MB-231 tumors is consistent with previous reports of TNBC residual cell survival following chemotherapy and their subsequent reactivation leading to recurrence (*12*, *13*). Ex vivo transcriptomic studies of xenografts and PDX models showed that residual TNBC cells surviving chemotherapy experienced a metabolic shift from glycolysis to mitochondrial metabolism (*13*, *16*). Additionally, transcriptomes of primary and recurrent TNBC tumors were found to be very similar, while those of residual tumors showed distinct gene expression with altered metabolic profiles favoring a reliance on OXPHOS (*13*). There is well-established evidence in the literature that indicates that mechanisms underlying an up-regulation in mitochondrial metabolism are multifaceted. For example, up-regulation of OXPHOS following chemotherapy in TNBC xenografts was attributed to structural changes in the mitochondria mediated by mitochondrial fusion (*16*). PDX models of residual TNBC tumors treated with chemotherapy reported increased mitochondrial elongation and OXPHOS levels associated with the mitochondrial inner membrane fusion protein optic atrophy 1 (OPA 1). In a different study, in TNBC models including MDA-MB-231 cells, MYC and MCL1 were found to cooperatively promote paclitaxel-resistant cancer stem cells by increasing OXPHOS, reactive oxygen species (ROS) production, and *HIF-1*α expression (*28*). The leptin–Janus kinase (JAK)/signal transducer and activator of transcription 3 (STAT3) pathway was reported to increase fatty acid oxidation through the transcription of metabolic gene *CPT1B*, supporting cancer stemness and chemoresistance in multiple TNBC models including MDA-MB-231 cells treated with paclitaxel (*30*). Paclitaxel sensitivity in TNBC was found to induce endoplasmic reticulum stress by promoting the interaction between ring finger protein 5 (RNF5) and l-glutamine carrier protein SLC1A5, causing ubiquitination and degradation of SLC1A5, thus dysregulating levels of TCA cycle intermediates (*31*). Therefore, there are numerous mechanistic pathways underlying the observed up-regulation in mitochondrial metabolism during residual disease, highlighting the importance of functional imaging as it reflects molecular, cellular, and tissue-level dynamics.

Our imaging and metabolomic analysis in this article reflect a common downstream feature of the above-described different mechanistic studies. Through barcode-mediated clonal tracking, others have shown that chemotherapy-treated residual TNBC tumors maintained the subclonal architecture of untreated tumors, while their phenotypic metabolic profiles were distinct from untreated tumors (favoring an up-regulation in mitochondrial respiration as determined from seahorse and RNA-sequencing assays) (*13*). These observations suggest that residual TNBC tumors can adopt a transient state that is not mediated by clonal selection as a drug resistance mechanism. Lack of clonal selection in TNBC samples from patients after chemotherapy was further confirmed by the same group, as well as from other studies (*32*–*34*). However, clonal selection has been reported to accompany chemoresistance in a subset of TNBC patients (*33*). Our goal, therefore, is to develop imaging strategies that capture the downstream changes in metabolism that characterize resistance (Figs. 1 to 5). Gaining insights into the transient nature of metabolically distinct heterogeneous subpopulations can lead to therapeutic benefits if targeted during the chemotherapy-induced vulnerable phase (*35*, *36*).

We report substantial variability in mitochondrial membrane potential (TMRE_60_), which could be attributed to both differences within and between tumors. As illustrated by the scatterplots showing mean fluorescence within individual tumors (fig. S1), there is considerable variability in mitochondrial metabolism between resistant tumors at each treated time point over time compared to primary/untreated tumors. Intertumoral biological heterogeneity following chemotherapy has been extensively shown in TNBC and across cancer types (*22*, *37*). Additionally, as shown in Fig. 4A and fig. S3, intratumoral heterogeneity is a result of the metabolically distinct areas that emerge following therapy in MDA-MB-231 tumors. The reliance of metabolically distinct, drug-resistant tumor subpopulations on mitochondrial metabolism is being increasingly reported in the literature (*29*, *38*). Despite fluctuations seen in TMRE_60_ signal, we have focused on the broader trends that are consistently seen over time corresponding to clinically relevant stages. Both treated and untreated MDA-MB-231 tumors exhibited significant increases in TMRE_60_ at D8 compared to D0. The increase observed in untreated tumors is not surprising. As reported by our group and others, tumor volume can influence metabolism, with significant changes between large (D8) compared to medium (D0) tumors that are actively growing (*19*, *20*). Further, clustering analysis showed an increase in intratumoral metabolic heterogeneity over time in treated MDA-MB-231 tumors but not in untreated MDA-MB-231 tumors (fig. S6). Increases in fatty acid uptake (Bodipy_60_) were seen in untreated, actively growing tumors starting from D10. The increase in fatty acid uptake in untreated MDA-MB-231 tumors at D10 and D18 compared to D0 was not seen in treated mice over time. Together, our findings led us to conclude that increases in mitochondrial metabolism following paclitaxel treatment are not entirely fueled by fatty acids and may be driven by a nonglucose, nonfat metabolic source as well (potentially amino acids). This observation is supported by our metabolomic findings.

No enduring changes in metabolism or metabolic heterogeneity were observed in sensitive HCC-1806 tumors. A decrease in fatty acid uptake and an increase in glucose uptake were seen during acute treatment that were resolved at the time of residual disease, highlighting the transient nature of these metabolic alterations. The metabolic inflexibility of HCC-1806 tumors is consistent with previous studies showing that TNBC cells in the basal-like subtype (HCC-1806) tend to modulate their metabolism less than TNBC cells in the mesenchymal-like subtype (MDA-MB-231) (*39*). Day-matched resistant HCC-1806 tumors displayed elevated mitochondrial metabolism and intratumoral heterogeneity compared to day-matched sensitive HCC-1806 tumors, which has been reported to correlate with increased therapy resistance (*4*, *40*). While the increase in metabolic heterogeneity in day-matched resistant HCC-1806 tumors compared to sensitive HCC-1806 tumors was nonsignificant, this could be attributed to the small sample size of each group (*n* = 3). Because of the sporadic nature of recurrence in HCC-1806 tumors treated at the same volume, controlling the sample size of each group was challenging and beyond the scope of this study (*22*). Future work could delve further into metabolic adaptions within the same tumor line displaying heterogeneous treatment responses.

Our results show that increased mitochondrial metabolism is a defining feature of metabolic reprogramming in residual disease and recurrence in the models used. The extensive heterogeneity within TNBC underscores the need for a tool to visualize functional phenotypic changes that appear to be conserved across patients. Specifically, metabolic phenotyping and transcriptomics of patient biopsies revealed OXPHOS as the most up-regulated pathway in TNBC tumors (*41*, *42*). OXPHOS up-regulation after chemotherapy was further validated in residual TNBC PDX models (*13*), with studies showing a distinct transcriptome program pointing to elevated OXPHOS in micro-metastases that is conserved across PDX models (*43*).

Longitudinal imaging allowed for key time points representing the largest changes in metabolism to be selected for metabolomics. Consistent with our imaging studies, TCA cycle intermediates were significantly increased, while the glycolytic intermediate lactate was significantly decreased in residual MDA-MB-231 tumors, pointing to elevated NADH [reduced form of nicotinamide adenine dinucleotide (oxidized form)] levels, which suggests an increase in oxidative phenotypes during residual disease, possibly driven by fatty acid and amino acid metabolic sources. Lipid droplet formation has been reported to be up-regulated during chemoresistance in TNBC and has been postulated to be a prognostic marker of tumorigenicity (*14*, *44*, *45*). Our metabolomic analysis suggests an increase in lipid metabolism in residual MDA-MB-231 tumors, also consistent with our imaging studies that showed increases in fatty acid uptake at key time points of residual disease. However, imaging pinpointed the earliest time point at which significant metabolic changes were observed, a temporal phenomenon that would not have been possible to capture with metabolomics only due to the destructive nature of the technique. Metabolomics also suggested an increase in other fuel sources, specifically amino acids, consistent with studies that highlight the reliance of treatment-resistant MDA-MB-231 cells on amino acids including serine (*46*, *47*). This was not possible to capture with our current imaging platform only. Except for citrate, metabolic shifts were not as pronounced in residual disease of HCC-1806 tumors, supporting our in vivo findings. Through in vivo, temporal metabolic phenotyping coupled with metabolomic analysis at key time points, our results underscore the importance of complementarily using both approaches in studying evolution-based therapy resistance.

Our work demonstrates the need to capture temporal metabolic profiles during treatment. Shifts from the primary tumor along each metabolic axis occur at different time points for MDA-MB-231 and HCC-1806 tumors. For example, MDA-MB-231 tumors showed a significant increase in mitochondrial metabolism as early as 2 days after the third paclitaxel dose; similarly, a significant change in glucose and fatty acid uptake occurred as early as 2 days after the fifth paclitaxel dose. A significant change in fatty acid uptake was observed in HCC-1806 tumors 2 days after the third paclitaxel dose. Additionally, metabolic shifts were transient in the latter. Such dynamic metabolic profiles are supported by previous in vitro TNBC studies (*35*, *36*), highlighting the need to identify the time points of reversible, temporary metabolic changes of TNBC tumors to optimize treatment efficacy (*13*).

Work by our group and others has shown how metabolic multiplexing, i.e., the holistic consideration of several statistically significant variables, enables a better understanding of tumor metabolism (*48*–*52*). Metabolic multiplexing in this work permitted the relationship between multiple metabolic pathways to be visualized throughout a tumor’s transition from the primary tumor to regression to residual disease to recurrence. In addition, analyses of shifts in intratumoral metabolic heterogeneity using multiplexed endpoints identified the emergence of metabolically distinct spatial clusters in resistant MDA-MB-231 tumors following paclitaxel treatment, specifically subpopulations that rely on glucose uptake, mitochondrial metabolism, or both.

The use of immunocompromised mice is a limitation of our study given that immune cells, specifically tumor-infiltrating lymphocytes, can affect tumor responses to chemotherapy (*53*, *54*). Further, the TNBC models used were limited to studying local recurrences only, and further studies investigating metabolic reprogramming at distant metastatic sites will be informative. Additionally, the use of window chambers is a weakness of our models since these restrict the imaged penetration depth due to tissue turbidity (*55*). To confirm the reliability of our model in the best way possible, we have performed day-matched imaging (window chamber) and metabolomics (no window chamber) in paired cohorts of mice on both MDA-MB-231 and HCC-1806 tumors (Fig. 3). Consistency across imaging and metabolomic results supports the metabolic features imaged in the window chambers, which increases our confidence in the interpretation of the results. This work demonstrates that tracking dynamic metabolic changes and heterogeneity are important markers for comparing tumor responses to chemotherapy. Assessing these temporal, quantitative changes has the potential to inform on both the risk of tumor recurrence and the approach to tailor therapies to target specific vulnerabilities of residual subpopulations to reduce or delay the onset of tumor recurrence.

## MATERIALS AND METHODS

### Experimental design

Optical metabolic imaging coupled with metabolomics was used to characterize in vivo dynamic changes along three major metabolic axes throughout paclitaxel treatment, tumor regression, residual disease, and recurrence in paclitaxel-sensitive and paclitaxel-resistant tumor models.

### Cell culture

All cell lines used in this study were purchased from the Duke Cell Culture Core Facility (CCF), where they were subjected to mycoplasma testing [American Type Culture Collection (ATCC) catalog no. HTB-26 for MDA-MB-231 tumor line and ATCC catalog no. CRL-2335 for HCC-1806 tumor line]. MDA-MB-231 cells were cultured in minimum essential medium (MEM) (Gibco 11095080), supplemented with 10% fetal bovine serum (Gibco A3160501), 1% antibiotics (penicillin-streptomycin; Gibco 15140122), 1% nonessential amino acids (Gibco 11140050), and 1% sodium pyruvate (Gibco 11360070). HCC-1806 cells were cultured in ATCC-modified RPMI 1640 (Gibco A1049101), supplemented with 10% fetal bovine serum (Gibco A3160501), and 1% antibiotics (penicillin-streptomycin; Gibco 15140122). Cells were passaged at ~80% confluency. All the cells were incubated at 37°C with 5% CO_2_ and 95% relative humidity.

### Animal studies

All murine experimental protocols were approved by the Duke University Institutional Animal Care and Use Committee (IACUC) (protocol A038-21-02). Mice were housed in an on-site, pathogen-free facility with ad libitum access to food and water with standard light/dark cycles. Murine experiments were performed under isoflurane gas anesthesia, and all efforts were made to minimize suffering.

### Xenograft models

To establish xenograft models, the abdomen of 6- to 8-week-old athymic nude females (Charles River, strain no. 490) was cleaned using 70% ethanol. Then, the fourth right mammary gland was palpated and injected with 100 μl of cell suspension using a 27-gauge needle. For injection, cells (MDA-MB-231 or HCC-1806) were washed with sterile phosphate-buffered saline (PBS) twice and resuspended in phenol red–free 50% Matrigel (Corning) in PBS at 5 × 10^6^ cells/100 μl.

Two-dimensional tumor measurements were made by calipers twice a week, and the tumor volume was calculated according to the following formula: volume = (short diameter^2^) × (long diameter/2). When the tumor volume reached ~150 mm^3^, mice were divided into two groups. One group of mice was left untreated. The second group of mice was treated with paclitaxel according to a standard therapy regimen, 20 mg/kg intraperitoneally, every other day for 10 days. Paclitaxel used in this study was purchased from the Duke out-patient pharmacy (Athenex, vehicle: polyoxyl, castor oil, dehydrated alcohol, and citric acid) and protected from light.

### Murine mammary window chamber

Titanium window chambers, 12 mm in diameter with No. 2 glass coverslips, were surgically implanted over the fourth right mammary gland of female athymic nude (Charles River, strain no. 490) mice using an established procedure (*56*) at key time points of the tumor’s therapeutic life cycle. Tumors modeled either a primary tumor (*n* = 10), an early regressing tumor (during active paclitaxel treatment, *n* = 10), a late regressing tumor (immediately following paclitaxel withdrawal, *n* = 10), residual disease (minimum tumor volume, no active growth, *n* = 10), or a recurrent tumor [actively growing in volume, size of the regrown tumor is back to the size of the primary tumor (~150 mm^3^), *n* = 13 for MDA-MB-231 mice]. Mice were allowed >24 hours to recover from surgery before imaging experiments. All intravital microscopy studies with treated mice were carried out in two independent trials.

### In vivo metabolic imaging

To ensure a normalized metabolic rate in each animal, mice were fasted for 4 hours (water provided) before each imaging session (*57*). Following fasting, blood glucose levels were measured to ensure that they were below 100 mg/dl. During imaging, mice were anesthetized using isoflurane (1 to 1.5%, v/v) in room air in an induction chamber. Then, the animal was transferred to a heated microscope stage with isoflurane supply where a background fluorescence image (laser on) and background noise image (laser off) were acquired before probe administration to account for the signal from sources other than the fluorescent indicators.

For in vivo administration, TMRE (Life Technologies/Thermo Fisher Scientific) was diluted to a final concentration of 75 μM in sterile PBS, 2-NBDG (Thermo Fisher Scientific) was diluted to a final concentration of 6 mM in sterile PBS, and Bodipy FL C16 (Thermo Fisher Scientific) was diluted in sterile PBS to a final concentration of 200 μM, according to previously established protocols (*17*). For simultaneous imaging with TMRE and 2-NBDG probes, 100 μl of 75 μM TMRE was injected first, followed by 100 μl of 6 mM 2-NBDG 20 min later. For Bodipy FL C16 imaging, 100 μl of 200 μM Bodipy FL C16 was injected 2 days following TMRE/2-NBDG imaging to provide sufficient clearance time for the first set of metabolic fluorophores while still capturing all metabolic endpoints in the same animal. All probes were delivered systemically via retro-orbital injection. Imaging was performed every 2 days, alternating between probe sequences until the end of the viability of the imaging window or the tumor burden limit to capture dynamic metabolic changes. TMRE, 2-NBDG, and Bodipy FL C16 images were acquired 60 min following their respective injection (termed TMRE_60_, 2-NBDG_60_, and Bodipy_60_, respectively).

To account for daily light source variation, all images were background-subtracted and calibrated according to a Rhodamine B standard imaged at each imaging session before image analysis.

### Fluorescence microscopy system

A custom-built, previously characterized, wide-field fluorescent microscope (*17*) was used to capture all metabolic endpoints. This system uses a Nikon CFI E Plan Achromat 4× objective [numerical aperture (NA) = 0.1, Nikon Instruments Inc., USA] to create a single frame field of view of 2.1 mm × 1.6 mm and a lateral resolution of 2.2 μm, as measured using a 1951 USAF resolution target (*17*). This microscope is controlled by a custom-designed LabVIEW software.

For TMRE imaging, a 555-nm crystal laser (CL555-100-O, CrystaLaser, Reno, NV, USA) was used for excitation, followed by a 573-nm long pass dichroic mirror (FF573-Di01-25 × 36, Semrock, Rochester, New York, USA) to reject the excitation light in the emission channel. The emission signal was collected using a liquid crystal tunable filter (LCTF) (VariSpec VIS-7-35, PerkinElmer Inc. Waltham, MA, USA, 7-nm bandwidth) at 585 nm and a high-resolution dual-modal charge-coupled device (CCD) (ORCA-Flash 4.0, Hamamatsu, Japan). An exposure time of 5 s was used to capture TMRE fluorescence.

For 2-NBDG imaging, a 488-nm crystal laser (DL488–100-O, CrystaLaser, Reno, NV, USA) was used for excitation, followed by a 505-nm long pass dichroic mirror (DMLP505R, Thorlabs, USA) to reject the excitation light in the emission channel. The emission signal was collected using the previously described LCTF (programmed to collect at 545 nm) and CCD. An exposure time of 2 s was used to capture 2-NBDG fluorescence.

For Bodipy FL C16 imaging, a 488-nm crystal laser (DL488–100-O, CrystaLaser, Reno, NV, USA) was used for excitation, followed by a 505-nm long pass dichroic mirror (DMLP505R, Thorlabs, USA) to reject the excitation light in the emission channel. The emission signal was collected using the previously described LCTF (programmed to collect at 515 nm) and CCD. An exposure time of 5 s was used to capture Bodipy FL C16 fluorescence.

### Optical imaging data processing and statistical analysis

All postprocessing image analyses were performed using MATLAB (MathWorks, USA). Before further analysis, each image collected at the 60-min time point was subtracted by the average value of an image collected with the laser source off (dark noise) and by the average signal imaged before fluorescent probe injection (pre-injection image). The resulting images were used for all analyses, displayed images, and statistical comparisons in this study. The aspect ratio of representative images shown was modified to enhance the display. Ridge plots were selected to visualize the changes in fluorescence intensity distribution across whole images over time for each metabolic endpoint. Statistical comparisons for changes in metabolic probe uptake were performed using a KS statistical test with blocked permutations (*n* = 1000 random permutations per test) to compare distributions, before binning data for graphing (*58*).

### Intratumoral metabolic heterogeneity analyses

**Fig. 6.**
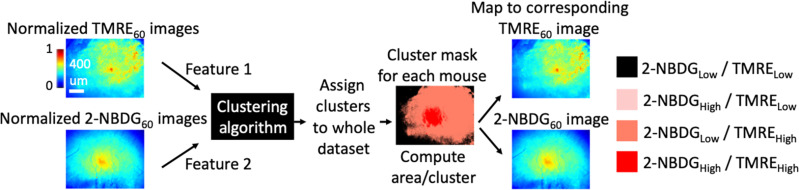
Approach for manual clustering for analyzing intratumoral metabolic heterogeneity. Schematic of clustering analysis using TMRE_60_ and 2-NBDG_60_ endpoints to visualize spatial clusters within individual images. Manual clustering was performed using mean probe uptakes as cutoff values for assignment into four major clusters.

TMRE_60_ and 2-NBDG_60_ intensities for all pixels across all mice were clustered using a manual clustering algorithm for both MDA-MB-231 and HCC-1806 tumors at all imaged time points (Fig. 6). We defined four metabolic clusters: low TMRE_60_/low 2-NBDG_60_ [2-NBDG_Low_/TMRE_Low_], high TMRE_60_/high 2-NBDG_60_ [2-NBDG_High_/TMRE_High_], low 2-NBDG_60_/high TMRE_60_ [2-NBDG_Low_/TMRE_High_], and high 2-NBDG_60_/low TMRE_60_ [2-NBDG_High_/TMRE_Low_]. Cluster assignment was performed on a pixel-by-pixel basis across all images corresponding to all mice at each key time point for both cell lines (all the available pixels across all mice). The mean values of each metabolic indicator were used as cutoffs for segmentation into high and low clusters. Area fraction or cluster distribution was calculated for each cluster as a percentage of the total number of clusters. Low TMRE_60_/low 2-NBDG_60_ (2-NBDG_Low_/TMRE_Low_) clusters were ignored in this calculation as these corresponded primarily to pixels from the background noise or vasculature. Area fractions were compared across time points and across cell lines using a one-way ANOVA followed by Tukey’s post hoc test for multiple comparisons.

### Metabolomic and statistical analyses

Mammary tumors were harvested from paired cohorts of mice at each time point (mice that were injected with metabolic probes were not used for metabolomic experiments), flash-frozen, and pulverized to a powder (*n* = 5 tumors per group). Tumor harvest for metabolomics was day-matched across tumor lines to enable comparisons. Tumors were collected at D0 and D24 to correspond to primary and residual tumors that escaped therapy, respectively. Samples were analyzed for amino acids, acylcarnitines, and organic acids using a stable isotope dilution technique. For amino acids and acylcarnitine measurements, samples were prepared as previously described (*59*, *60*) and levels were quantified using flow injection tandem mass spectrometry using a Waters TQD mass spectrometer equipped with an Acquity UPLC system and controlled by the MassLynx 4.1 operating system (Waters, Milford, MA). Organic acids were quantified as previously described (*61*) using Trace Ultra GC coupled to ISQ MS operating under Xcalibur 2.2 (Thermo Fisher Scientific, Austin, TX). Assays were completed by Duke Molecular Physiology Institute’s Metabolomics Core Laboratory.

Data were analyzed as previously described using the R POMA package; there was no data filtering, and data were normalized by the sum of all metabolites per sample, log-transformed, and underwent Pareto scaling (*62*). Comparisons of metabolites between time points are displayed as volcano plots, where |Log_2_FoldChange| > 0.05 and *P* values (*t* test) < 0.05 were considered significant.

### Histological and immunohistochemical staining

Mammary tumors were harvested at each time point, fixed with 4% paraformaldehyde in PBS, and stained for hematoxylin and eosin or ATP5A1 (PA5-25704, Thermo Fisher Scientific, 1:1500). Staining was performed by the Duke Pathology Research Laboratory Core Facility. Analysis was performed by a board-certified veterinary pathologist.
