# Supplementary material for: Optical imaging reveals chemotherapy-induced metabolic reprogramming of residual disease and recurrence
Source: Sci Adv. 2024 Apr 5;10(14):eadj7540. doi: 10.1126/sciadv.adj7540 (PMC10997195; doi:10.1126/sciadv.adj7540)
Supplement: Supplementary file 1 — Figs. S1 to S7 [file sciadv.adj7540_sm.pdf]

Science Advances
AAAS

Supplementary Materials for

Optical imaging reveals chemotherapy-induced metabolic reprogramming of residual disease and recurrence

Enakshi D. Sunassee *et al.*

Corresponding author: Enakshi D. Sunassee, es364@duke.edu

Sci. Adv. **10**, eadj7540 (2024)
DOI: 10.1126/sciadv.adj7540

This PDF file includes:

Figs. S1 to S7

Supplementary Materials

<u>Supplementary Figure S1:</u>

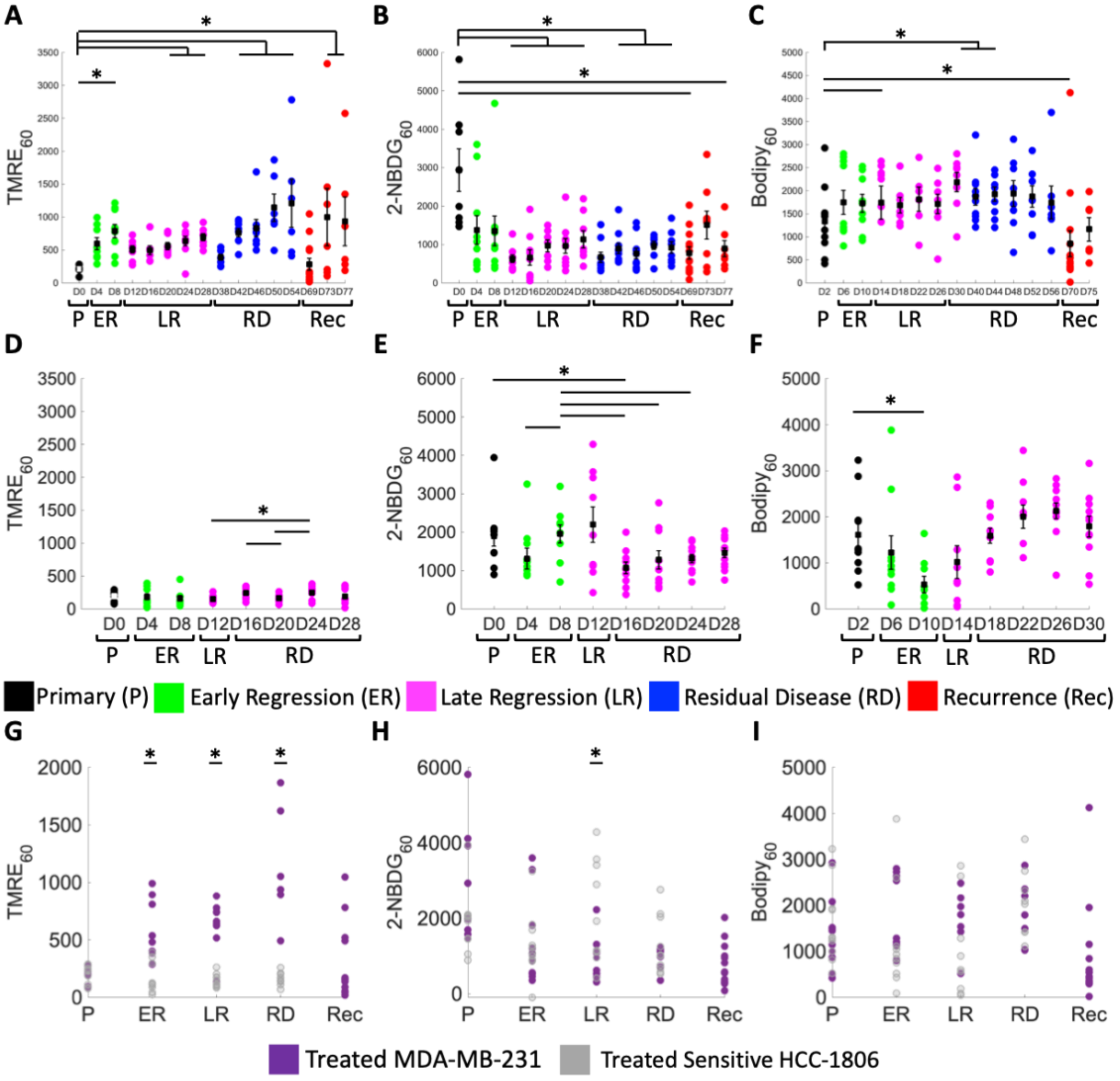

Supplementary Fig. S1: Unlike sensitive HCC-1806 tumors, resistant MDA-MB-231 tumors show increased mitochondrial metabolism and decrease use of glucose over their therapeutic lifecycle. Scatter plots of individual MDA-MB-231 tumors showing changes in **A)** mitochondrial metabolism (TMRE$_{60}$), **B)** glucose uptake (2-NBDG$_{60}$), and **C)** fatty acid uptake (Bodipy$_{60}$) at the primary (P) (n=10), early regressing (ER) (n=10), late regressing (LR) (n=10), residual disease (RD) (n=10), and recurring (Rec) (n=13) stages. Scatter plots of individual HCC-1806 tumors showing changes in **D)** mitochondrial metabolism, **E)** glucose uptake and **F)** fatty acid uptake at the primary (P) (n=10), early regressing (ER) (n=10), late regressing (LR) (n=10), and residual disease (RD) (n=10) stages. Scatter plots of individual MDA-MB-231 and HCC-1806 tumors showing changes in **G)** mitochondrial metabolism, **H)** glucose uptake and **I)** fatty acid uptake at the primary (P) (n=10), early regressing (ER) (n=10), late regressing (LR) (n=10), and residual disease (RD) HCC-1806 and MDA-MB-231 tumors, and recurring (Rec) MDA-MB-231 tumors (n=13). Statistical analyses were performed on all pixels at each time point using a

Kolmogorov-Smirnov (KS) test. An asterisk, “*”, signifies statistical significance between two time points joined by the corresponding line.

Supplementary Figure S2:

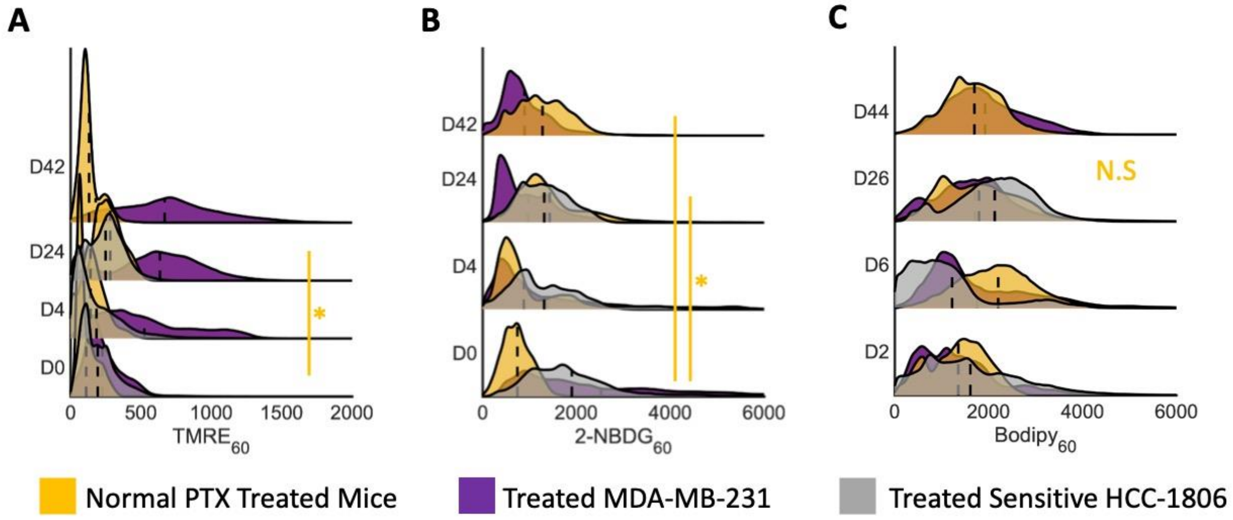

Supplementary Fig. S2: Minimal metabolic changes are reported in normal athymic nudes treated with paclitaxel. Ridge plots of probe uptake across all pixels and all mice at each time point for **A)** mitochondrial metabolism ($TMRE_{60}$), **B)** glucose uptake ($2\text{-}NBDG_{60}$), and **C)** fatty acid uptake ($Bodipy_{60}$) for normal athymic nudes treated with paclitaxel (n=5 per probe), MDA-MB-231 mice treated with paclitaxel (n=10 per probe), and HCC-1806 mice treated with paclitaxel (n=10 per probe). Statistical differences in probe uptake over time are shown for normal athymic nudes treated with paclitaxel and were determined using a Kolmogorov-Smirnov (KS) test. Vertical dashed lines superimposed on each curve correspond to the average fluorescence.

Supplementary Figure S3:

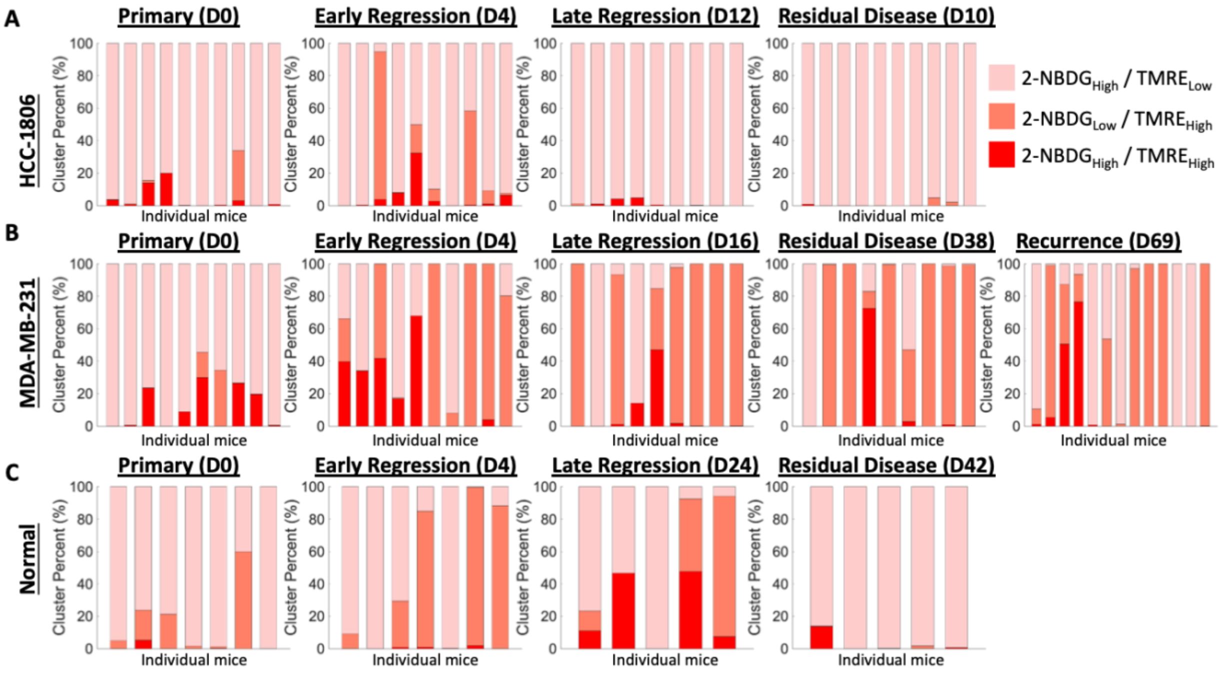

Supplementary Fig. S3: Resistant MDA-MB-231 tumors show increased intra-tumoral metabolic heterogeneity following treatment compared to their primary tumor; compared to sensitive HCC-1806 tumors; and compared to treated normal mice. Bar graphs showing changes in area fraction (cluster percent (%)) of cluster distributions corresponding to [2-NBDG$_{High}$/TMRE$_{Low}$] clusters, [2-NBDG$_{High}$/TMRE$_{High}$] clusters, or [2-NBDG$_{Low}$/TMRE$_{High}$] clusters across primary, early regression, late regression, residual disease, and recurrence (if applicable) for **A)** treated HCC-1806 tumors (n=10), **B)** treated MDA-MB-231 tumors (n=10), **C)** treated normal mice (n=7) on a per-mouse basis.

<u>Supplementary Figure S4:</u>

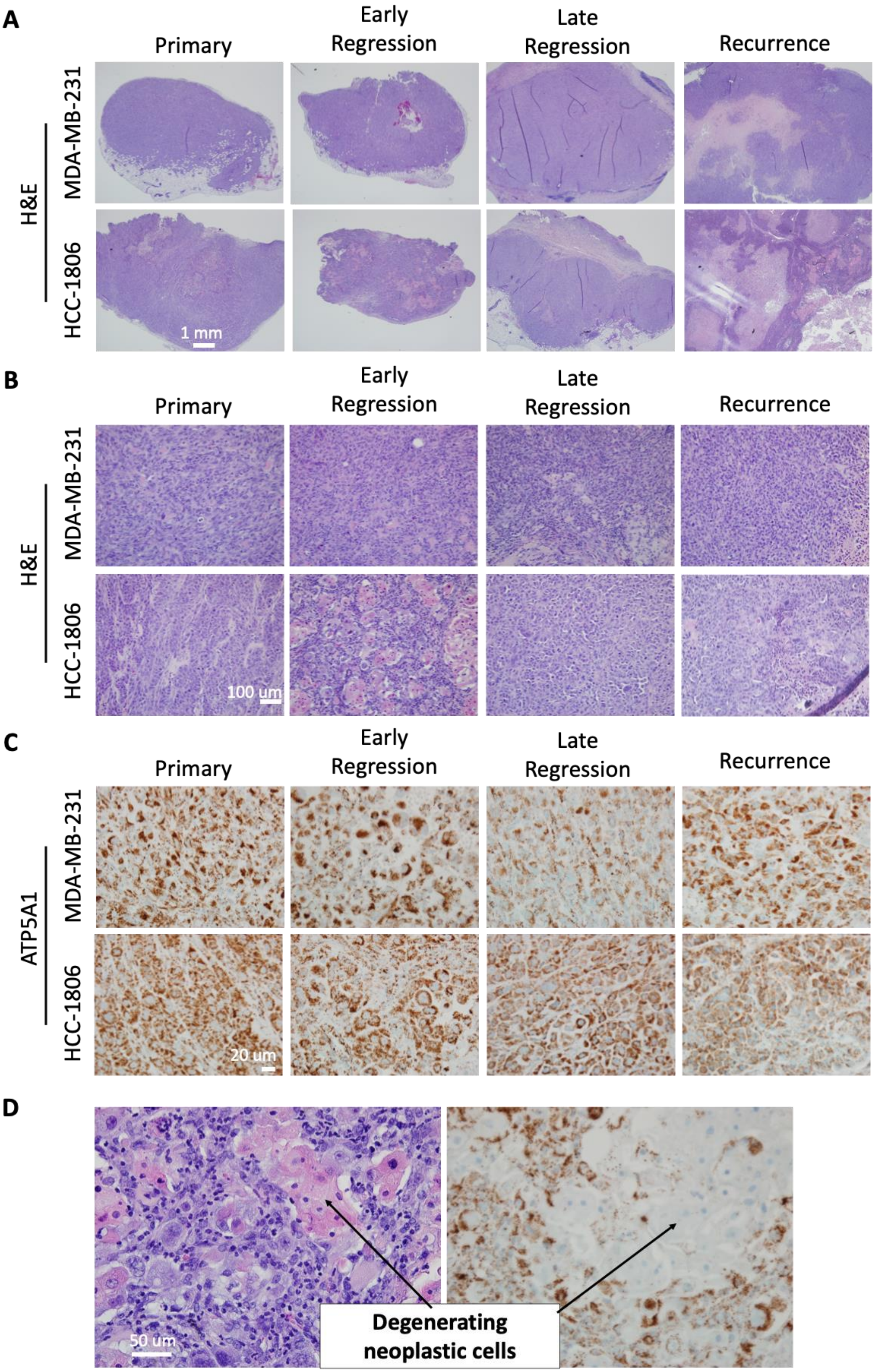
A
Primary
Early Regression
Late Regression
Recurrence
H&E
MDA-MB-231
HCC-1806
1 mm
B
Primary
Early Regression
Late Regression
Recurrence
H&E
MDA-MB-231
HCC-1806
100 um
C
Primary
Early Regression
Late Regression
Recurrence
ATP5A1
MDA-MB-231
HCC-1806
20 um
D
50 um
Degenerating neoplastic cells

Supplementary Fig. S4: Histological analysis confirms viability of primary, regressing, residual, and recurrent tumors and immunohistochemistry shows mitochondrial staining in viable tumor regions only. A) Representative Hematoxylin and Eosin staining of primary (n=4), early regressing (n=4), late regressing (n=5), and recurrent (n=5) MDA-MB-231 tumors (20X magnification). Representative Hematoxylin and Eosin staining of primary (n=4), early regressing (n=4), late regressing (n=6), and recurrent (n=4) HCC-1806 tumors (20X magnification). **B)** Representative Hematoxylin and Eosin staining of primary, early regressing, late regressing, and recurrent MDA-MB-231 and HCC-1806 tumors (200X magnification). **C)** Immunohistochemistry with mitochondria stained with ATP5A1 were imaged and quantified from sectioned primary, early regressing, late regressing, and recurrent MDA-MB-231 and HCC-1806 tumors (600X magnification, focusing on viable tumor sections). **D)** 600X Hematoxylin and Eosin and ATP5A1 staining confirm ATP5A1 staining in viable tumor cells only. Increased necrosis seen in rapidly growing resistant HCC-1806 tumors is expected, particularly since these were harvested at a relatively large tumor burden, after imaging was complete.

Supplementary Figure S5:

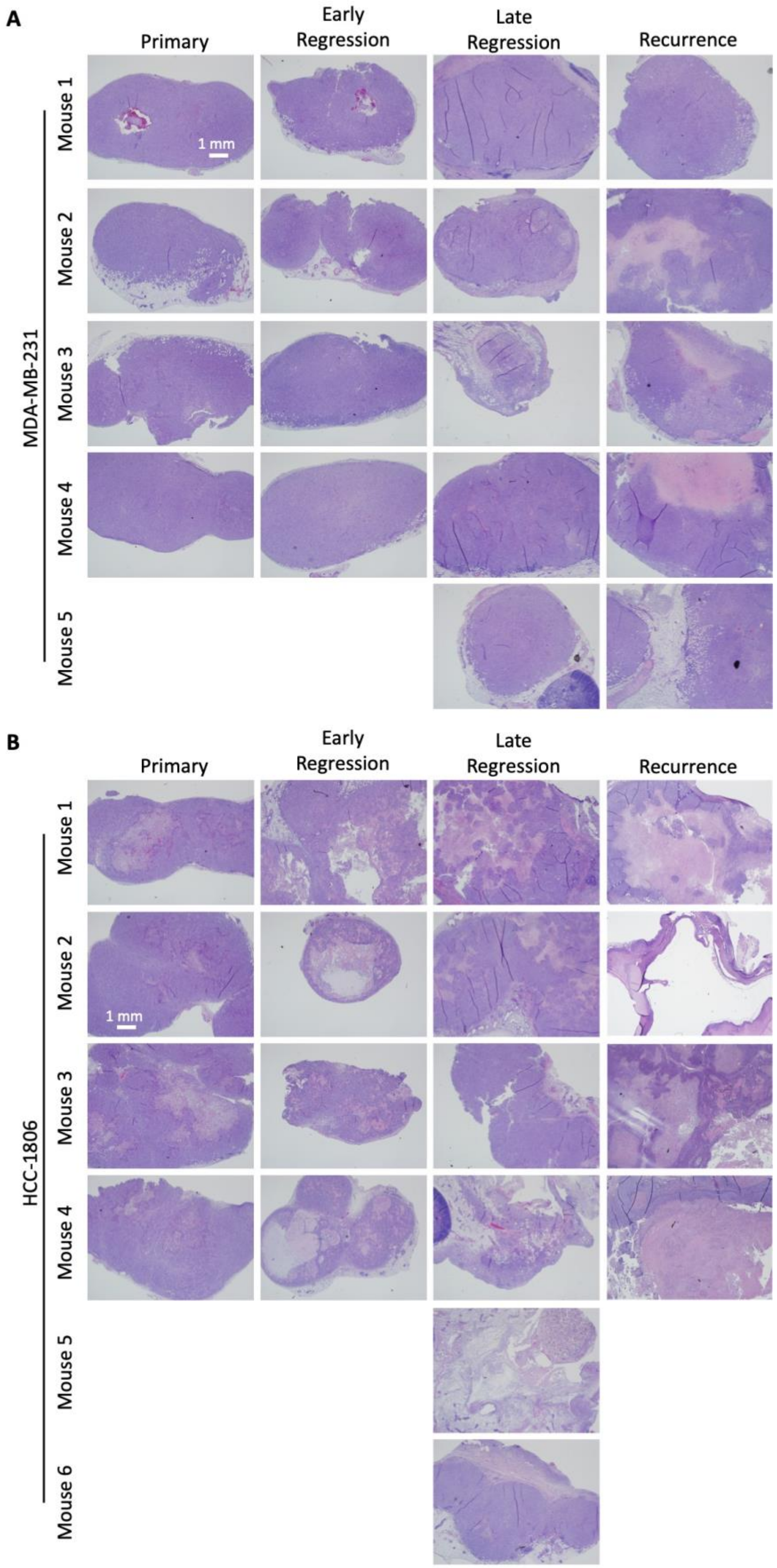

Supplementary Fig. S5: Histological analysis confirms viability of primary, regressing, residual, and recurrent tumors. A) Hematoxylin and Eosin staining of all primary (n=4),

early regressing (n=4), late regressing (n=5), and recurrent (n=5) MDA-MB-231 tumors (20X magnification). **B)** Hematoxylin and Eosin staining of all primary (n=4), early regressing (n=4), late regressing (n=6), and recurrent (n=4) HCC-1806 tumors (20X magnification). Increased necrosis seen in rapidly growing resistant HCC-1806 tumors is expected, particularly since these were harvested at a relatively large tumor burden, after imaging was complete.

Supplementary Figure S6:

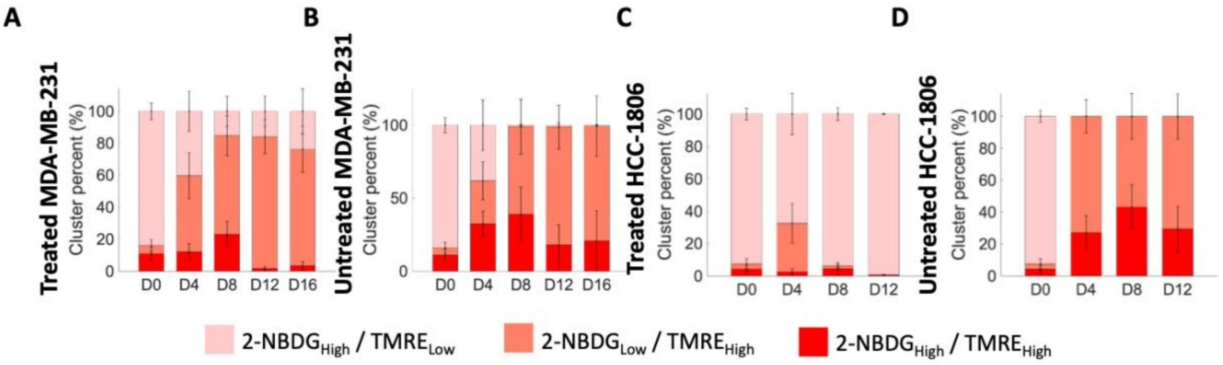

Supplementary Fig. S6: MDA-MB-231 tumors show significantly increased intra-tumoral metabolic heterogeneity following treatment compared to untreated counterparts. Bar graphs showing changes in area fraction (cluster percent (%)) of cluster distributions corresponding to [2-NBDG$_{High}$/TMRE$_{Low}$] clusters, [2-NBDG$_{High}$/TMRE$_{High}$] clusters, or [2-NBDG$_{Low}$/TMRE$_{High}$] clusters across **A)** treated MDA-MB-231 tumors (n=10), **B)** untreated MDA-MB-231 tumors (n=5), **C)** treated HCC-1806 tumors (n=10), **D)** untreated HCC-1806 tumors (n=5). Statistical differences in cluster percent across days were determined using a one-way ANOVA followed by Tukey's post hoc test.

Supplementary Figure S7:

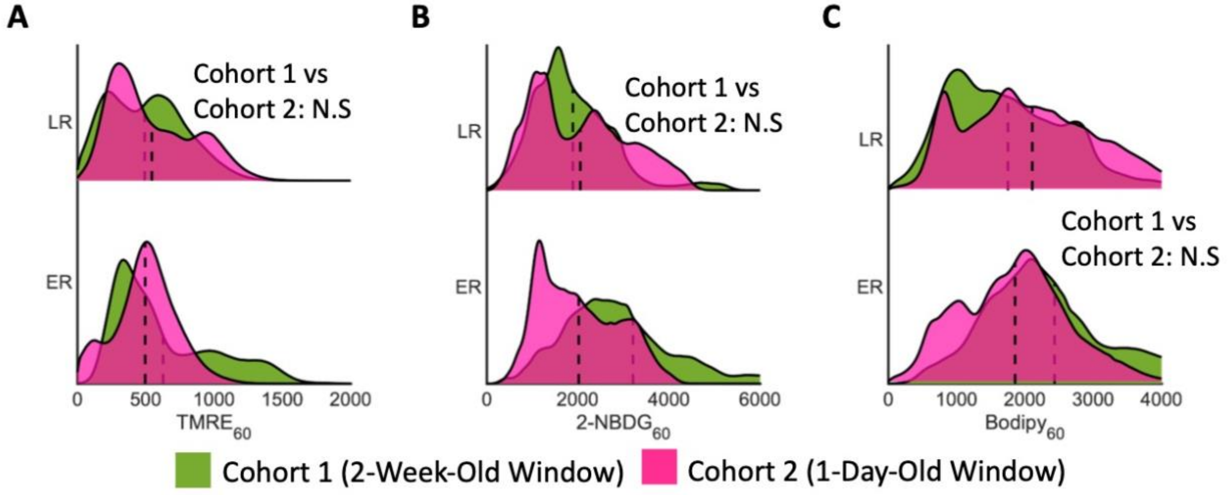

Supplementary Fig. S7: Age of the window chamber does not affect fluorescent intensities. Ridge plots of probe uptake across all pixels and all mice at the Early Regression (ER) and Late Regression (LR) time point for **A)** mitochondrial metabolism (TMRE$_{60}$), **B)** glucose uptake (2-NBDG$_{60}$), and **C)** fatty acid uptake (Bodipy$_{60}$). Statistical differences in probe uptakes were determined using a Kolmogorov-Smirnov (KS) test. Vertical dashed lines superimposed on each curve correspond to the average fluorescence.
